# Structural brain development between childhood and adulthood: Convergence across four longitudinal samples

**DOI:** 10.1016/j.neuroimage.2016.07.044

**Published:** 2016-11-01

**Authors:** Kathryn L. Mills, Anne-Lise Goddings, Megan M. Herting, Rosa Meuwese, Sarah-Jayne Blakemore, Eveline A. Crone, Ronald E. Dahl, Berna Güroğlu, Armin Raznahan, Elizabeth R. Sowell, Christian K. Tamnes

**Affiliations:** a Department of Psychology, University of Oregon, Eugene, OR, USA; b Center for Translational Neuroscience, University of Oregon, Eugene, OR, USA; c Institute of Child Health, University College London, London, UK; d Department of Pediatrics, Keck School of Medicine at USC/Children's Hospital of Los Angeles, Los Angeles, CA, USA; e Institute of Psychology, Leiden University, Leiden, The Netherlands; f Leiden Institute for Brain and Cognition, Leiden University, Leiden, The Netherlands; g Institute of Cognitive Neuroscience, University College London, London, UK; h Institute of Human Development, University of California Berkeley, Berkeley, CA, USA; i Child Psychiatry Branch, National Institute of Mental Health, Bethesda, MD, USA; j Research Group for Lifespan Changes in Brain and Cognition, Department of Psychology, University of Oslo, Oslo, Norway

**Keywords:** Adolescence, Cerebral cortex, MRI, Replication, Sex differences, White matter

## Abstract

Longitudinal studies including brain measures acquired through magnetic resonance imaging (MRI) have enabled population models of human brain development, crucial for our understanding of typical development as well as neurodevelopmental disorders. Brain development in the first two decades generally involves early cortical grey matter volume (CGMV) increases followed by decreases, and monotonic increases in cerebral white matter volume (CWMV). However, inconsistencies regarding the precise developmental trajectories call into question the comparability of samples. This issue can be addressed by conducting a comprehensive study across multiple datasets from diverse populations. Here, we present replicable models for gross structural brain development between childhood and adulthood (ages 8–30 years) by repeating analyses in four separate longitudinal samples (391 participants; 852 scans). In addition, we address how accounting for global measures of cranial/brain size affect these developmental trajectories. First, we found evidence for continued development of both intracranial volume (ICV) and whole brain volume (WBV) through adolescence, albeit following distinct trajectories. Second, our results indicate that CGMV is at its highest in childhood, decreasing steadily through the second decade with deceleration in the third decade, while CWMV increases until mid-to-late adolescence before decelerating. Importantly, we show that accounting for cranial/brain size affects models of regional brain development, particularly with respect to sex differences. Our results increase confidence in our knowledge of the pattern of brain changes during adolescence, reduce concerns about discrepancies across samples, and suggest some best practices for statistical control of cranial volume and brain size in future studies.

## Introduction

The human brain continues to develop structurally between childhood and adulthood, as evident from longitudinal studies using structural MRI (Aubert-Broche et al., 2013, Dennison et al., 2013, Ducharme et al., 2015, Lebel and Beaulieu, 2011, Lenroot et al., 2007, Sowell et al., 2004, Tamnes et al., 2013, Urošević et al., 2012, Vijayakumar et al., 2016, Wierenga et al., 2014b). Many of these studies report similar overall changes, but substantial inconsistencies in the developmental trajectories of structural brain measures have also been noted in previous reports (Ducharme et al., 2015, Mills and Tamnes, 2014, Walhovd et al., 2016). While the potential impact of quality control procedures (Ducharme et al., 2015), or software used to estimate brain measures (Walhovd et al., 2016), on structural brain developmental trajectories have been investigated, no study has yet attempted to replicate developmental trajectories across multiple longitudinal samples. As accurate population models of human brain development are crucial for our understanding of typical development as well as neurodevelopmental disorders, it is essential that our models are replicable across diverse samples.

Characterizing the developmental trajectories of gross brain structures is essential not only for understanding basic processes of brain development, but also for informed analysis considerations. Comparative structural MRI studies of brain development are often confronted with the question as to whether to “normalize” brain measures by controlling for differences in cranial or brain size – intracranial volume (ICV) or whole brain volume (WBV) – between participants (O'Brien et al., 2011). This is an important consideration for studies describing changes in specific brain structures across development, to ensure that observed regional effects are independent of global size changes. By controlling for cranial or brain size, researchers can be more confident that the differences observed between participants (or across time) are not due to overall cranial or brain size differences between individuals (or over time), but instead reflect differences in the specific structure of interest (Sanfilipo et al., 2004). It is not clear from the available literature whether absolute changes in regional brain volumes, or changes in these structures relative to cranial/brain size, are more important and relevant for the understanding of the developing brain. This may be particularly important to ascertain for volumes of structures that do not directly correlate with cranial or brain size, where the decision whether or not to correct for cranial or brain size in the analyses can affect both the results and their interpretation (O'Brien et al., 2011).

The present study analyzed four separate datasets collected in three different countries in an attempt to replicate gross brain developmental trajectories. Using a team science approach and open collaboration framework to improve replication, the aim of this study was to test two simple but fundamental questions that are highly relevant and yet unresolved issues in the developmental neuroimaging field: 1) How do gross brain volumes develop between childhood and early adulthood? 2) How does accounting for global measures of ICV or WBV affect developmental trajectories?

To address the first of our two questions, we focused on characterizing how ICV and WBV as well as gross regional brain volumes, namely cortical grey matter volume and cortical white matter volume, change across development in each of our longitudinal samples. In order to control for potential confounds that could be introduced by differences in automated software (Walhovd et al., 2016), or quality control procedures (Ducharme et al., 2015), we processed, quality-controlled, and analyzed these four datasets using the same methods. Controlling for these factors ensured we could more confidently assess the potential impact of sample differences and certain statistical decisions on these developmental models. We hypothesized that both ICV, WBV, and regional brain volumes would show continued development through adolescence, and that, having standardized the analysis methods, there would be broad similarities in the developmental trajectories seen across the four samples.

To investigate our second question, we examined how controlling for ICV or WBV affects the developmental trajectories of two major regional brain measures: cortical grey matter volume (CGMV) and cerebral white matter volume (CWMV). We assessed the effects of controlling for ICV or WBV on the developmental trajectories of these brain volumes using two different methods previously used in the published literature: (i) *the proportional method*: where the regional brain volume of interest is divided by ICV or WBV leaving a proportional value and (ii) *the covariate method*: where shared variance with ICV or WBV is accounted for by regression statistics through the inclusion of ICV or WBV as a covariate in the developmental model. These two methods of controlling for total cranial/brain size were applied to the age-only developmental models as well as models incorporating age and sex variables to characterize what can happen to developmental trajectories and sex comparisons when investigations use these methods, as has been done previously in the aging and disease literature (Pintzka et al., 2015, Sanfilipo et al., 2004). Given our first hypothesis that ICV and WBV would show dynamic changes across this time-period, we hypothesized that incorporating ICV or WBV using the proportional or the covariate method would have differing effects on the modelled trajectories of our regions of interest. We further expected that incorporating measures of ICV or WBV in models incorporating sex would modulate the effect of sex on model fit, since many of the sex differences seen in regional brain volumes are thought to be attributed to differences in boys having, on average, larger brain volumes as compared to girls (Giedd et al., 2012).

## Material and methods

### Participants

This study utilized four separate longitudinal datasets collected from four separate sites spanning three countries (Norway, the Netherlands, and the United States). These samples were drawn from the University of Oslo (Neurocognitive Development; NCD), Leiden University (Braintime), National Institute of Mental Health (NIH Child Psychiatry Branch; CPB) and University of Pittsburgh. Demographic characteristics for each sample are described in Table 1. Details regarding participant recruitment at each site are described in Supplemental material.

### Image processing

Details regarding image acquisition at each site are described in Supplemental material. Two samples (CPB and NCD) were scanned using 1.5-T MRI machines, and two samples (Pittsburgh and Braintime) were scanned using 3-T MRI machines. MRI processing was performed with the FreeSurfer 5.3 image analysis suite, which is documented and freely available online (http://surfer.nmr.mgh.harvard.edu/). The technical details of these procedures are described in detail in seminal publications (Dale et al., 1999, Fischl et al., 1999, Fischl et al., 2002). This processing stream includes motion correction (Reuter et al., 2010), removal of non-brain tissue using a hybrid watershed/surface deformation procedure (Ségonne et al., 2004), automated Talairach transformation, non-parametric non-uniform intensity normalization (Sled et al., 1998), tessellation of the grey/white matter boundary, automated topology correction (Fischl et al., 2001, Ségonne et al., 2007), and surface deformation following intensity gradients to optimally place the grey/white and grey/cerebrospinal fluid borders at the location where the greatest shift in intensity defines the transition to the other tissue class (Dale et al., 1999, Dale and Sereno, 1993, Fischl and Dale, 2000). Each cortical model was registered to a spherical atlas using individual cortical folding patterns to match cortical geometry across participants (Dale et al., 1999).

Images were then processed using FreeSurfer 5.3's longitudinal stream (Reuter et al., 2012). This process includes the creation of an unbiased within-subject template space and image using robust, inverse consistent registration (Reuter et al., 2010). Several processing steps, such as skull stripping, Talairach transforms, atlas registration as well as spherical surface maps and parcellations are then initialized with common information from the within-subject template, significantly increasing reliability and statistical power (Reuter et al., 2012). Each of the datasets was processed on workstations and operating systems at their respective universities (see Supplemental methods). While it has been shown that different operating systems and workstations can impact FreeSurfer's brain volume estimates (Gronenschild et al., 2012), these effects are generally small and it is not known whether they affect longitudinal change trajectories.

### Brain measures of interest

Measures of brain structure were acquired for each participant at each time-point. For the purposes of this study, we obtained measures of intracranial volume (ICV), whole brain volume (WBV), cortical grey matter volume (CGMV) and cerebral white matter volume (CWMV). As FreeSurfer 5.3's longitudinal pipeline assumes a constant ICV (by averaging ICV across all timepoints), the ICV measures for the current study were extracted from each scan after being processed through the cross-sectional pipeline, but before being processed through the longitudinal pipeline. Since ICV correlates with the determinant of the transform matrix used to align an image with an atlas, FreeSurfer uses an atlas-based spatial normalization procedure to estimate ICV (Buckner et al., 2004). WBV was a composite measure of white matter and grey matter including the cerebellum. CGMV was measured using the surface-based reconstructed image, and CWMV was measured as the volume inside the white surface less anything that is not considered white matter.

### Analysis procedure

The first aim of this study was to characterize how cranial volume and brain size (ICV and WBV) and gross regional brain volumes (CGMV and CWMV) change across development in four separate longitudinal samples. For this aim, we used mixed-effects modeling (R version “Wooden Christmas-Tree”; nlme package version 3.1-126). This analysis method allows an estimation of the fixed effects of measured variables on volume change, while incorporating the longitudinal nature of the data by including within-person variation as nested random effects. We chose to assess polynomial models rather than spline modeling in order to make our methods more compatible with previously conducted studies assessing brain developmental trajectories, and to provide more straightforward interpretation of our model comparisons. The following age models were tested:

1. Linear model: Volume = Intercept + α(age)
2. Quadratic model: Volume = Intercept + α(age) + β(age^2^)
3. Cubic model: Volume = Intercept + α(age) + β(age^2^) + γ(age^3^).

where α, β, and γ represent the constant terms defining the effects of each fixed term. All models included a random intercept for each participant. Models were compared to determine which was the best-fit using likelihood ratio tests and Akaike Information Criterion (AIC). Specifically, the model with the lowest AIC value that was also significantly different (as determined by the likelihood ratio test) than the less complex models was selected. All models were tested against a baseline (null) model that included only the random effects (i.e., the intercept for each participant), but not the fixed effects of interest.

The second aim of this study was to demonstrate how controlling for ICV or WBV size could impact the results and interpretations of investigations into brain development. We examined how controlling for ICV or WBV influences the best fitting age model for CGMV and CWMV using the two most commonly used methods for normalizing brain measures in both the developmental and aging literature: the proportional method and the covariate method. To test the proportional method, CGMV or CWMV was divided by ICV or WBV for each participant at each time point. We then assessed the best fitting age model for these adjusted volumes using AIC and likelihood ratio tests in the same manner as described for the unadjusted volumes. To test the covariate method, we included ICV or WBV as a fixed term in the three age models described above, and assessed the best fitting models using AIC and likelihood ratio tests in the same manner as described for the unadjusted volumes.

Finally, we investigated how controlling for ICV or WBV impacts observed differences in grey and white matter volumes between females and males across the developmental period studied. To do so, we examined whether adding sex as a fixed term improved the fit of the developmental trajectories for the i) best fit models for raw volumes, ii) best fit models for adjusted volumes, or iii) best fit covariate models. For each method we compared the best fitting age models (as determined using the methods described above) against the same models with the added sex term. For example, if the best fit age model for raw volumes were quadratic, we would compare this model to one with sex added as a fixed term:

1. Quadratic model: Volume = Intercept + α(age) + β(age^2^)
2. Sex differences model: Volume = Intercept + α(age) + β(age^2^) + γ(sex).

Our study did not examine the effects of sex on overall developmental shape, as we assumed the developmental patterns for CGMV and CWMV to be similar between sexes, as has reported in previous studies (Aubert-Broche et al., 2013, Tamnes et al., 2013, Wierenga et al., 2014a).

Our analysis script is publicly available on the Open Science Framework: https://osf.io/9saj5/.

## Results

### Gross developmental trajectories using raw volumes

**Fig. 1:**
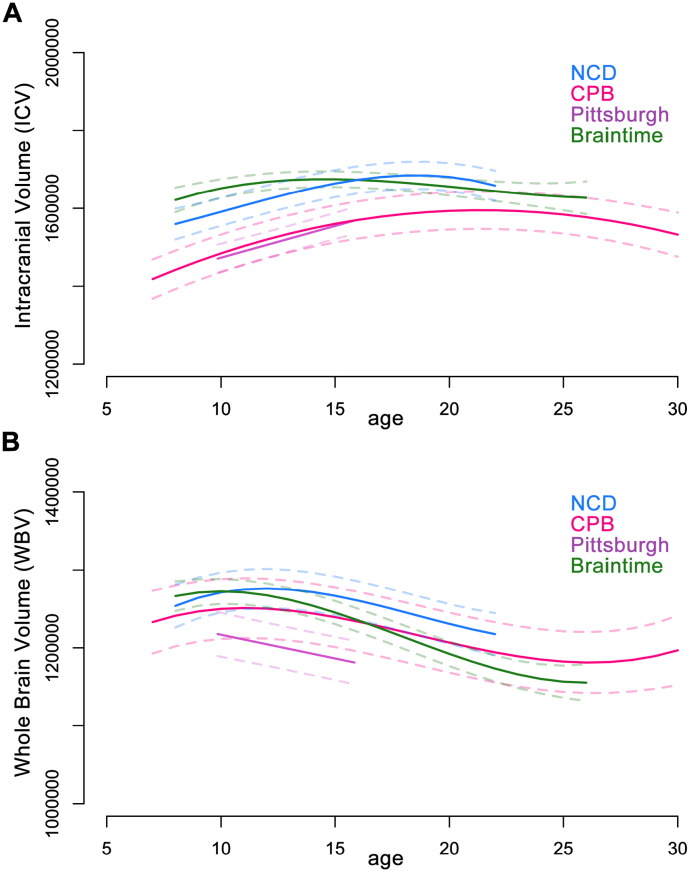
Best fitting age models for [A] ICV and [B] WBV. The best fitting models are represented by the solid lines. Dashed lines represent 95% confidence intervals. Age in years is measured along the x-axis and brain measure in mm^3^ along the y-axis. ICV: intracranial volume; WBV: whole brain volume; CPB: Child Psychiatry Branch; NCD: Neurocognitive Development. See also Table S1 and Fig. S2.

In each of our four samples, both ICV and WBV showed substantial changes in volume between late childhood and early adulthood (Fig. 1; Table S1). These two measures, however, evinced distinct developmental trajectories.

#### Intracranial volume (ICV)

Nonlinear models were the best fit for ICV in three of the samples, whereas a linear model best fit ICV in the Pittsburgh sample (Table S1). Three of the population models suggest an annual increase of ~ 1% in ICV between late childhood and mid-adolescence (Figs. 1A and S2a), with one population model (Braintime) showing a more attenuated development of ICV in contrast to the others. Based on the three samples that extend into young adulthood, it appears that ICV begins to stabilize in late adolescence.

#### Whole brain volume (WBV)

Nonlinear models were the best fit for WBV in three of the samples, whereas a linear model best fit WBV in the Pittsburgh sample (Table S1). WBV followed a different developmental pattern than ICV, with all samples showing a decrease in WBV across adolescence (Figs. 1B and S2b). However, it is unclear when the reduction begins, as two samples displayed a decrease in WBV between late childhood and mid-adolescence (Braintime and Pittsburgh), whereas the other two showed relative stability in this period (NCD and CPB). Based on the three samples that extend into young adulthood, it appears that WBV begins to stabilize in the early twenties. These results provide strong evidence that ICV and WBV continue to change between adolescence and adulthood, albeit following distinct trajectories.

#### Cortical grey matter volume (CGMV)

**Fig. 2:**
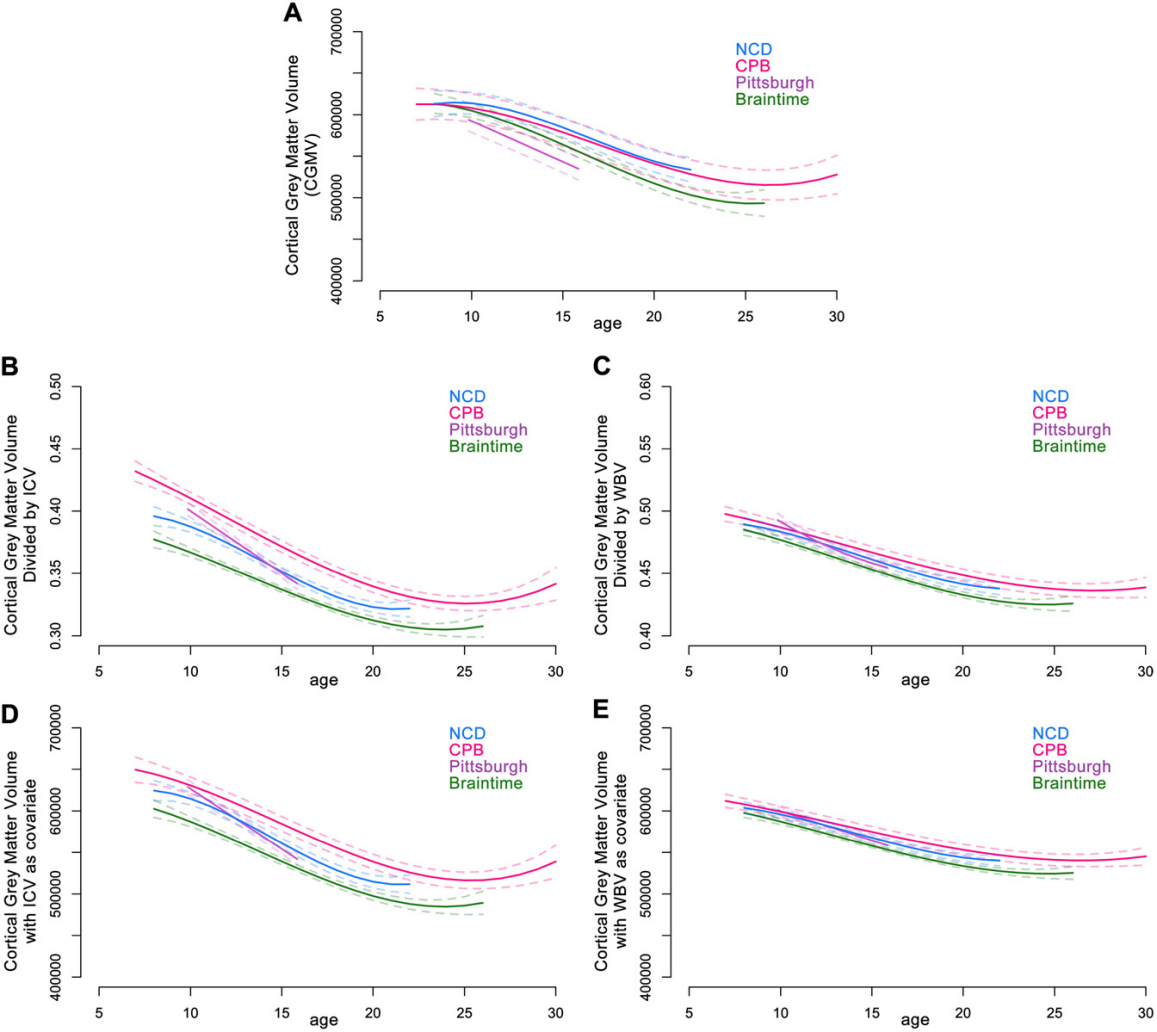
Best fitting age models for cortical grey matter volume (CGMV). Age in years is measured along the x-axis and brain measure along the y-axis. A. Raw values (mm^3^); B. CGMV adjusted by ICV (proportion); C. CGMV adjusted by WBV (proportion); D. CGMV with ICV included as a covariate (mm^3^); E. CGMV with WBV included as a covariate (mm^3^). Best fitting models are represented by the solid lines. Dashed lines represent 95% confidence intervals. ICV: intracranial volume; WBV: whole brain volume; CPB: Child Psychiatry Branch; NCD: Neurocognitive Development. See also Tables S2, S3, S5 and Figs. S1, S3.

Nonlinear models were the best fit for CGMV for three of the samples, whereas a linear model best fit CGMV in the Pittsburgh sample (Table S2). CGMV followed a similar trajectory in each of the four samples, with the models for two samples (NCD and CPB) almost entirely overlapping (Figs. 2A and S3a). The three samples that cover late childhood showed the same highest average CGMV estimate of ~ 615,000 mm^3^ around the youngest age investigated, at approximately 8 years of age. Overall, these models suggest that CGMV is highest in childhood, decreases through the second decade, and begins to stabilize in the third decade.

#### Cerebral white matter volume (CWMV)

**Fig. 3:**
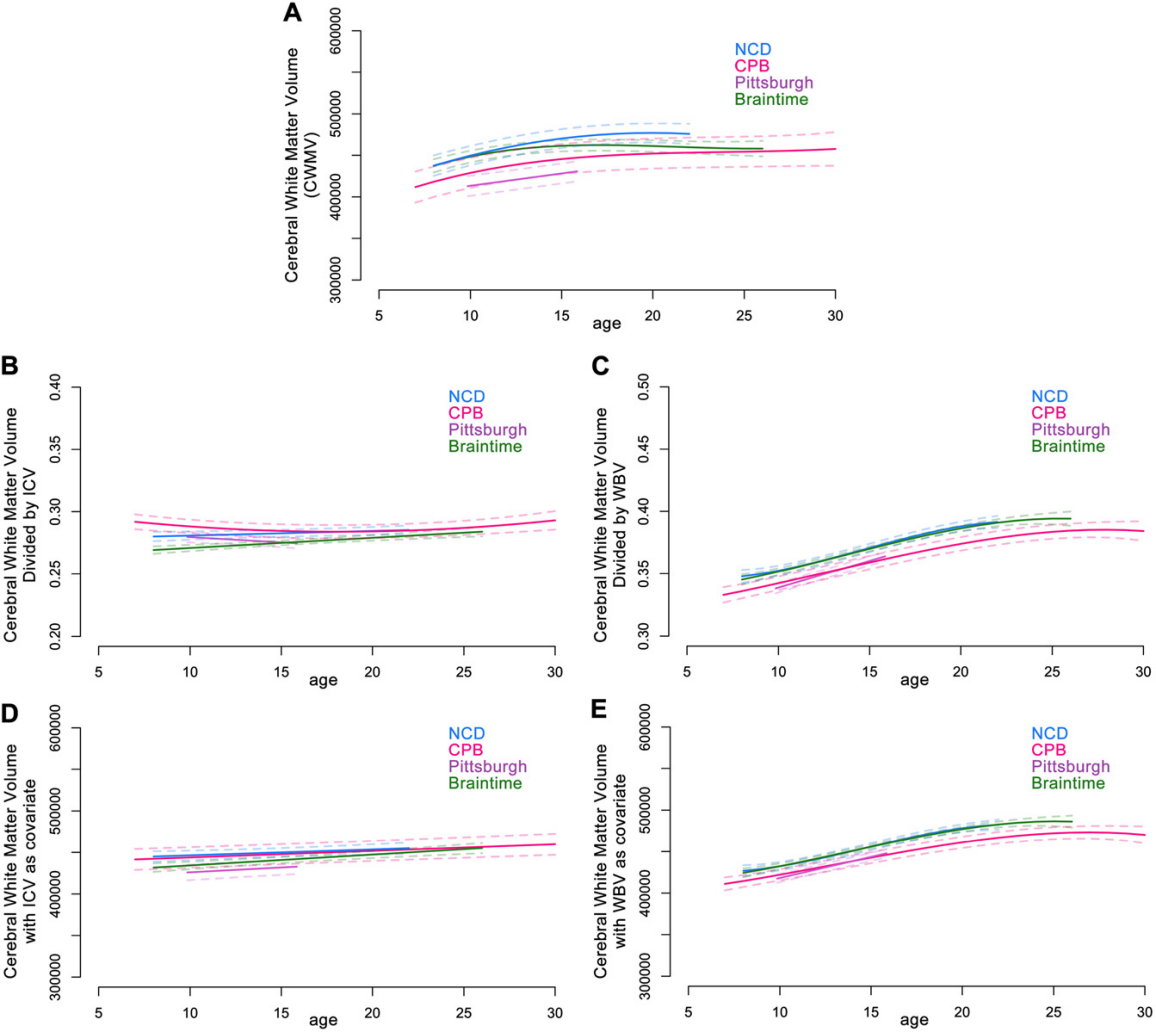
Best fitting age models for cerebral white matter volume (CWMV). Age in years is measured along the x-axis and brain measure along the y-axis. A. Raw values (mm^3^); B. CWMV adjusted by ICV (proportion); C. CWMV adjusted by WBV (proportion); D. CWMV with ICV included as a covariate (mm^3^); E. CWMV with WBV included as a covariate (mm^3^). Best fitting models are represented by the solid lines. Dashed lines represent 95% confidence intervals. ICV: intracranial volume; WBV: whole brain volume; CPB: Child Psychiatry Branch; NCD: Neurocognitive Development. See also Tables S2, S4, S6 and Figs. S1, S4.

Nonlinear models were the best fit for CWMV for three of the samples, whereas a linear model best fit CWMV in the Pittsburgh sample (Table S3). CWMV followed an overall similar trajectory in each of the four samples, showing an increase between late childhood and mid-adolescence (Figs. 3A and S4a). However, samples varied as to when cerebral white matter began to stabilize in volume. While each sample showed increases in CWMV until mid-adolescence, the models for the Pittsburgh, CPB, and NCD samples continued to show an increase in volume until late adolescence. Unlike CGMV, which was roughly of similar size across samples, the best fit models demonstrate that CWMV for the two samples drawn from the United States (CPB and Pittsburgh) tended to be smaller than the CWMV of the European samples (Braintime and NCD).

### Controlling for cranial/brain size

#### The proportional method

Using the proportional method (where a participant's CGMV or CWMV was divided by ICV or WBV), the best fitting models for CGMV adjusted by ICV were nonlinear for three of the samples, and linear for the Pittsburgh sample (Table S3); similar to the models for unadjusted volumes. However, the best fitting models for CGMV adjusted by WBV were nonlinear for all four samples (Table S3). Adjusting CGMV by either ICV or WBV using the proportional method narrowed the confidence intervals for the population models and shifted the developmental trajectories, so that CGMV no longer appeared relatively stable in late childhood (Fig. 2B and C). Adjusting CGMV by ICV amplified developmental changes, whereas adjusting CGMV by WBV attenuated changes (Fig. S3b and c). These models again show the appearance of an almost linear decrease in CGMV from late childhood until the early twenties.

In contrast, the trajectories for CWMV adjusted for cranial/brain size using the proportional method differed depending on the measure of cranial/brain size used. The best fitting models for CWMV adjusted by ICV were linear for three of the samples, and quadratic for the CPB sample (Table S4), while the best fitting models for CWMV adjusted by WBV were nonlinear for three of the samples, and linear for the Pittsburgh sample (Table S4). When CWMV was adjusted by ICV, the developmental trajectories were flattened considerably (Figs. 3B and S4b). When CWMV was adjusted by WBV, the developmental trajectories became more pronounced, with the increase in proportional white matter volumes continuing for longer than observed in the raw volumes (Figs. 3C and S4c). Further, when CWMV was adjusted by WBV, the models for the two US samples (CPB & Pittsburgh) overlapped, as did the models for the two European samples (NCD and Braintime), suggesting this method has the potential to control for population factors that could be unrelated to brain developmental trajectories.

#### The covariate method

Similar to both the raw and proportional volumes, the best fitting models for CGMV when either ICV or WBV is included as a covariate were nonlinear for three of the samples, and linear for the Pittsburgh sample (Table S5). As with the proportional method, including ICV as a covariate amplified the developmental changes seen in CGMV compared to using the raw values alone (Figs. 2D and S3d), and including WBV as a covariate attenuated the changes seen (Figs. 2E and S3e).

Like with the proportional method, the impact of including a covariate in the CWMV development models differed depending on the measure of cranial/brain size used. The best fitting models for CWMV when ICV is included as a covariate were linear for all of the samples (Table S6). However, similar to the raw and adjusted volumes, the models for CWMV when WBV is included as a covariate were nonlinear for three of the samples, and linear for the Pittsburgh sample (Table S6). Including ICV as a covariate in the model attenuated developmental changes in CWMV (Figs. 3D and S4d) to a greater extent than using the proportional method. Adding WBV as a covariate affected the developmental trajectory of CWMV similarly to the proportional method (Figs. 3E and S4e).

### Effects of controlling for cranial/brain size on perceived sex differences

Finally, we examined whether the overall brain volumes of CGMV and CWMV differed between females and males in our examined age ranges, and whether controlling for cranial/brain size affected the perceived differences between these groups. Across samples and regions, adding sex improved the model fit for each of the volumes of interest (ICV, WBV, CGMV and CWMV; Tables S1 and S2), suggesting that females and males show overall differences in size for each of these raw volume measures.

#### The proportional method

The inclusion of sex as a main effect in our models of proportional brain volumes (the proportional method) did not improve any of the model fits (*p*'s ≥ 0.05) (Tables S3 and S4). These results suggest that using the proportional method to adjust regional brain measures by ICV or WBV potentially removes the main effect of sex from developmental trajectories spanning the age ranges of the four samples.

#### The covariate method

The inclusion of sex as a main effect in the models including ICV or WBV as a covariate improved model fits in some cases (Tables S5 and S6). For all but one dataset (NCD), adding sex improved the age model of CGMV with an ICV covariate. However, the inclusion of sex in age models of CGMV while covarying for WBV only improved the model fit for one dataset (CPB). For CWMV, adding sex improved the age model of CWMV with an ICV covariate for all but the NCD dataset. The inclusion of sex in age models of CWMV with WBV as a covariate did not improve the model fits for any dataset. Overall, including ICV as a covariate when assessing the developmental trajectories of CGMV or CWMV does not account for the variance between females and males. However, including WBV as a covariate when assessing the developmental trajectories of CGMV or CWMV appears to account for variance between females and males. These results suggest that sex differences in developmental trajectories are less likely to be detected if WBV is used as a covariate than if ICV is used as a covariate in developmental models of gross regional brain volumes.

## Discussion

The purpose of this collaborative project was to examine how structural brain development patterns replicate across datasets and how these patterns are affected by different methods of accounting for global measures of brain size (ICV or WBV). Specifically, we adopted a team science approach in order to implement identical methods to examine these questions in four longitudinal MRI datasets in hopes of increasing our ability to report reliable findings for the field.

In contrast to early cross-sectional studies (Courchesne et al., 2000, Jernigan et al., 1991, Pfefferbaum et al., 1994), we present evidence for the continued development of both intracranial volume (ICV) and whole brain volume (WBV) into mid-to-late adolescence. While our results largely demonstrate convergence across samples in the developmental trajectory of ICV, samples drawn from the Netherlands and Norway (Braintime and NCD, respectively) displayed, on average, greater ICV than two samples from the United States (Pittsburgh and CPB). These differences likely reflect, in part, the average height difference between the sample populations (see Fig. S5), as the Dutch and Norwegian populations are among the tallest in the world (Cavelaars et al., 2000, Ogden et al., 2004, Schönbeck et al., 2013, Waaler, 1983). Our results also demonstrated that WBV followed a different developmental trajectory compared with ICV. This suggests that the two measures should not be treated interchangeably in developmental studies. While ICV increased until mid-to-late adolescence, WBV decreased in volume across adolescence. These results fill a gap in the lifespan model of WBV development presented in a meta-analysis of longitudinal studies (Fig. 3 in Hedman et al., 2012). With our results integrated into this lifespan model, it appears that WBV increases until some point between ages 10–15 years, then decreases until some point in the early twenties, after which it remains roughly stable until around age 40 when it begins to decrease again (Hedman et al., 2012).

Our finding, that CGMV is highest in childhood and decreases throughout adolescence, is in contrast to the popular narrative that cortical grey matter volume (CGMV) peaks around the onset of puberty (Giedd et al., 1999, Lenroot et al., 2007), but is in line with the results based on other longitudinal datasets not included in the current report (Aubert-Broche et al., 2013, Lebel and Beaulieu, 2011, Sowell et al., 2004, Wierenga et al., 2014b). Importantly, our results are also in accordance with evidence from histological studies. Early studies that described an adolescent peak in CGMV prompted some neuroimaging researchers to speculate that there could be a second wave of synaptogenesis in adolescence (Giedd et al., 1999, Lee et al., 2014). However, cellular and molecular evidence does not support this notion, as synaptic density plateaus between early childhood and puberty, even in relatively late maturing regions such as the prefrontal cortex (Huttenlocher and Dabholkar, 1997, Petanjek et al., 2011, Rakic et al., 1994, Webster et al., 2011). Despite the concomitant reduction in synaptic density and CGMV across adolescence, it is not possible to directly relate developmental changes in morphometric MRI measures to changes in cellular or synaptic anatomy (see Mills and Tamnes, 2014 for discussion). Multiple processes, including the encroachment of subcortical white matter and continued intracortical myelination, likely impact on measurements of CGMV by changing signal intensity values and contrasts (Grydeland et al., 2013, Westlye et al., 2010).

Our results for cerebral white matter volume (CWMV) are mostly similar to the findings of other longitudinal datasets not included in the current report (Aubert-Broche et al., 2013, Lebel and Beaulieu, 2011). However, these reports showed increasing CWMV throughout the second decade (Aubert-Broche et al., 2013, Lebel and Beaulieu, 2011), whereas the developmental models for our samples started showing relative stability in CWMV by mid-to-late adolescence.

The results of this study also have vital implications for the statistical analysis of structural brain development between late childhood and early adulthood. There is currently no consensus as to whether it is advisable to correct regional brain volumes by ICV or WBV in longitudinal studies of brain development, with some studies doing so (Herting et al., 2014, Urošević et al., 2012), and other studies opting to analyze the raw volumes of brain structures (Goddings et al., 2014, Raznahan et al., 2011, Wierenga et al., 2014a), and some studies reporting both (Dennison et al., 2013). In the current study, we assessed whether and how population age models for both CGMV and CWMV could be affected by controlling for these global measures of brain size. Our results suggest that, when included in the analyses, the specific shape of ICV or WBV development can change the shape of the age-curve on regional brain volumes. This impact was visible using either the covariate approach or the proportional approach. Different tissues and regions of the brain grow at different rates across the first three decades of life, as evident in the results of this study and other longitudinal developmental studies discussed within the present report. These different rates of development suggest that developmental studies should assess the developmental relationship (allometric scaling rules) between two MRI structural measurements (e.g. CGMV and ICV) before applying any correction procedures (O'Brien et al., 2011, Reardon et al., 2016). In addition, the current findings also suggest the metric one chooses to use to correct for overall brain size (i.e. ICV and WBV) is also important. In the present study, we found that brain volumes corrected for with WBV were more similar *across samples* in their developmental trajectory and overall size than brain volumes corrected with ICV, suggesting that WBV may be a more viable measure to use when correcting regional brain volumes in developmental studies. These results are congruent with others showing that the proportional method may be more susceptible to systematic error present in the ICV values (Sanfilipo et al., 2004). Specifically, when systematic error was introduced to the ICV values, the outcome of proportional brain volumes were “dramatically changed” whereas systematic error in ICV values did not affect the outcomes of the covariate method (Sanfilipo et al., 2004). Thus, it is thought that brain volumes corrected with the proportional method are more likely to vary across studies, which make them harder to directly compare against each other (Sanfilipo et al., 2004). As such, we recommend that future individual studies aim to display their initial understanding of how their overall brain size metric relates to their brain regions of interest as well as provide a clear hypothesis-driven approach during analytic and statistical testing.

As the role of sex in brain development is contentious, with some arguing that population-level sex differences in brain structure are due to population-level sex differences in physical size (Dekaban and Sadowsky, 1978; but see Lenroot et al., 2007, Sowell et al., 2007 for counter-argument), it is important to understand the relationship between commonly used covariates and sex in analyses of brain structure. Thus, in addition to demonstrating how controlling for ICV or WBV could affect the trajectory of population age models, this study also assessed how these methods affect sex differences across the age ranges studied. Specifically, the results suggest that the method in which one controls for ICV or WBV could affect observed sex differences in developing samples. When regional volumes were adjusted using the proportional method, sex was no longer a significant predictor of brain development. When using the covariate method, however, adding sex as a main effect showed mixed findings in terms of improving the model fit. Specifically, sex was only able to improve age models of regional brain volumes when ICV had been included as covariate. In contrast, when the age models included WBV as a covariate, sex was not able to account for any additional variance in all models except one. Therefore, it appears that using WBV as a covariate has the potential to absorb the variance in overall brain size between females and males on gross models of brain development spanning the second decade of life. In contrast, the proportional method suggests that sex differences in the overall size of gross brain volumes can be explained by sex differences in ICV or WBV. These two conflicting interpretations illustrate how methodological differences can impact on our understanding of brain development. At this time, the best way to determine the influence of sex on brain development may be to assess both raw and corrected brain measures (e.g., Dennison et al., 2013).

### Limitations

It is important to note that we took a polynomial modeling approach to the current project, which is subject to certain limitations pertinent to the interpretation of our replication project. For one, the age range of the sample will influence the best fitting model, as well as any inflection points (such as peaks and nadirs) in the model (Fjell et al., 2010, Mills and Tamnes, 2014). This is well illustrated in the current study, where many of the best model fits for the Pittsburgh study, which incorporated participants from a relatively narrow age range (10–16 years), were linear while models for the other samples were non-linear. Reviewing the models graphically (e.g. Fig. 1, Fig. 2), it can be seen that these linear and non-linear models describe very similar trajectories for the equivalent age ranges, and the different model fits predominantly reflect changing trajectories outside the overlapping age range. For this reason, deriving meaning solely from the “type” of model fit (i.e., cubic, quadratic, or linear) is not as meaningful when comparing samples of different age ranges. However, the patterns of change observed across each sample's age range are overall comparable, which illustrates the power of polynomial models to detect developmental changes in brain structure despite their relatively simple model structure.

Caution should be exerted in interpreting timing of peaks and the model predictions at the end-points of the age-trajectories from the quadratic and cubic models presented in the present paper, as global polynomial models have been shown to be substantially affected by irrelevant factors such as the age-range sampled (Fjell et al., 2010). Further, it should also be noted that this study investigated the effects of controlling for cranial/brain size on gross measures of cortical grey and cerebral white matter volumes. While not the focus of the present study, our post-hoc analysis of the prefrontal cortex found similar effects to the CGMV analysis (see Supplementary methods; S6 & S7). However, future studies applying these analysis methods to regional volumes are needed to further our understanding how accounting for cranial/brain size could affect the developmental trajectories of structures with distinct trajectories (e.g., subcortical structures).

## Conclusions

The present study examined four separate longitudinal datasets to characterize structural brain development. The results illustrate that ICV and WBV follow distinct developmental trajectories through adolescence. Further, our population models show that cortical grey matter is at its highest volume in childhood, decreases steadily through the second decade and shows decelerating decreases in the third decade, while cerebral white matter increases in volume until mid-to-late adolescence before showing relative stability. Finally, our results suggest that controlling for ICV or WBV impacts models of gross regional brain developmental trajectories and the perceived impact of sex on these models. These findings suggest that future developmental studies that do perform such corrections should provide the reader with: 1) the initial understanding of how the control variable (e.g. ICV or WBV) relates to the outcome of interest (e.g. CGMV, CWMV); 2) a clear rationale for the correction technique employed (e.g. wanting to look at relative, not absolute, sex differences); and 3) also preferably show the uncorrected results in addition to analyzing the relationships between the variable used for correction and age/other variables of interest.

## Author contributions

K.L.M., A.-L.G., M.M.H., R.M., and C.K.T. designed the present study, processed the data, conducted the analyses, and wrote the paper. S.-J.B., E.A.C., R.E.D., B.G., A.R., and E.R.S. designed and conducted the original studies, provided the data for the current study, funded the data collection and provided input into the design, interpretation and writing the manuscript.

## Figures and Tables

**Table 1:** Participant demographics for each sample. Mean (standard deviation), age and interval between scans are given in years. The table describes the total number of scans included in each sample, and the number of scans each study participant undertook (2–6 scans).

	NIH Child Psychiatry Branch			University of Pittsburgh		
	All	Female	Male	All	Female	Male
N	33	10	23	73	41	32
Age mean (SD)	15.8 (5.5)	16.6 (5.8)	15.4 (5.3)	13.3 (1.4)	12.9 (1.3)	13.9 (1.3)^a^
Age range	7.0–29.9	8.1–29.5	7.0–29.9	10.1–16.2	10.1–15.9	11.4–16.2
N scans	136	42	94	146	82	64
2 scans	–	–	–	73	41	32
3 scans	13	4	9	–	–	–
4 scans	7	2	5	–	–	–
5 scans	9	2	7	–	–	–
6 scans	4	2	2	–	–	–
Interval	4.1 (2.3)	4.1 (2.0)	4.0 (2.4)	2.2 (0.4)	2.2 (0.4)	2.1 (0.4)

	Neurocognitive Development			Braintime		

All	Female	Male	All	Female	Male

N	76	37	39	209	112	97
Age mean (SD)	15.2 (3.6)	15.1 (3.5)	15.4 (3.7)	15.7 (3.8)	15.5 (3.6)	15.9 (3.9)
Age range	8.2–21.9	8.4–21.8	8.2–21.9	8.0–26.6	8.2–24.8	8.0–26.6
N scans	152	74	78	418	224	194
2 scans	76	37	39	209	112	97
3 scans	–	–	–	–	–	–
4 scans	–	–	–	–	–	–
5 scans	–	–	–	–	–	–
6 scans	–	–	–	–	–	–
Interval	2.6 (0.2)	2.7 (0.2)	2.6 (0.2)	2.0 (0.1)	2.0 (0.1)	2.0 (0.1)

a Age difference between sexes (by design, see Supplementary material for details).

## Appendix A Supplementary data

Supplementary tables. Image 1

Supplementary material. Image 2
