# Supplementary material for: Structural brain development between childhood and adulthood: Convergence across four longitudinal samples
Source: Neuroimage. 2016 Nov 1;141:273–81. doi: 10.1016/j.neuroimage.2016.07.044 (PMC5035135; doi:10.1016/j.neuroimage.2016.07.044)
Supplement: Supplementary file 2 — Supplementary material. [file mmc2.docx]

**Supplemental Figures**

**
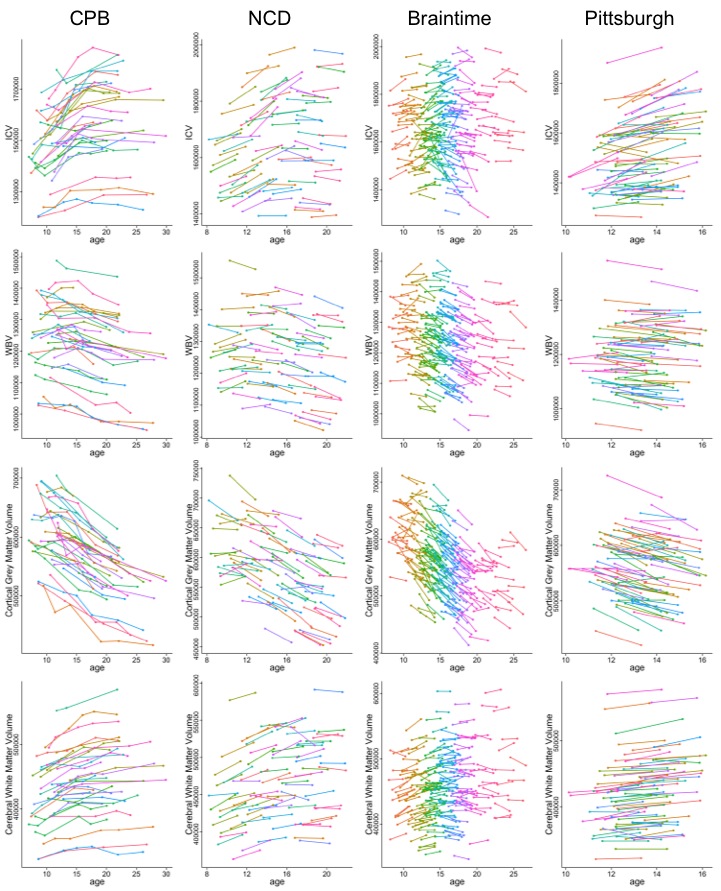
**

**Figure S1. Individual changes in ICV, WBV, CGMV, and CWMV across development.** Each line represents one participant, and each filled circle represents one time point. Age in years is measured along the x-axis and the raw value for each brain measure of interest (mm^3^) is along the y-axis. ICV: Intracranial Volume; WBV: Whole Brain Volume; CPB: Child Psychiatry Branch; NCD: Neurocognitive Development.

**
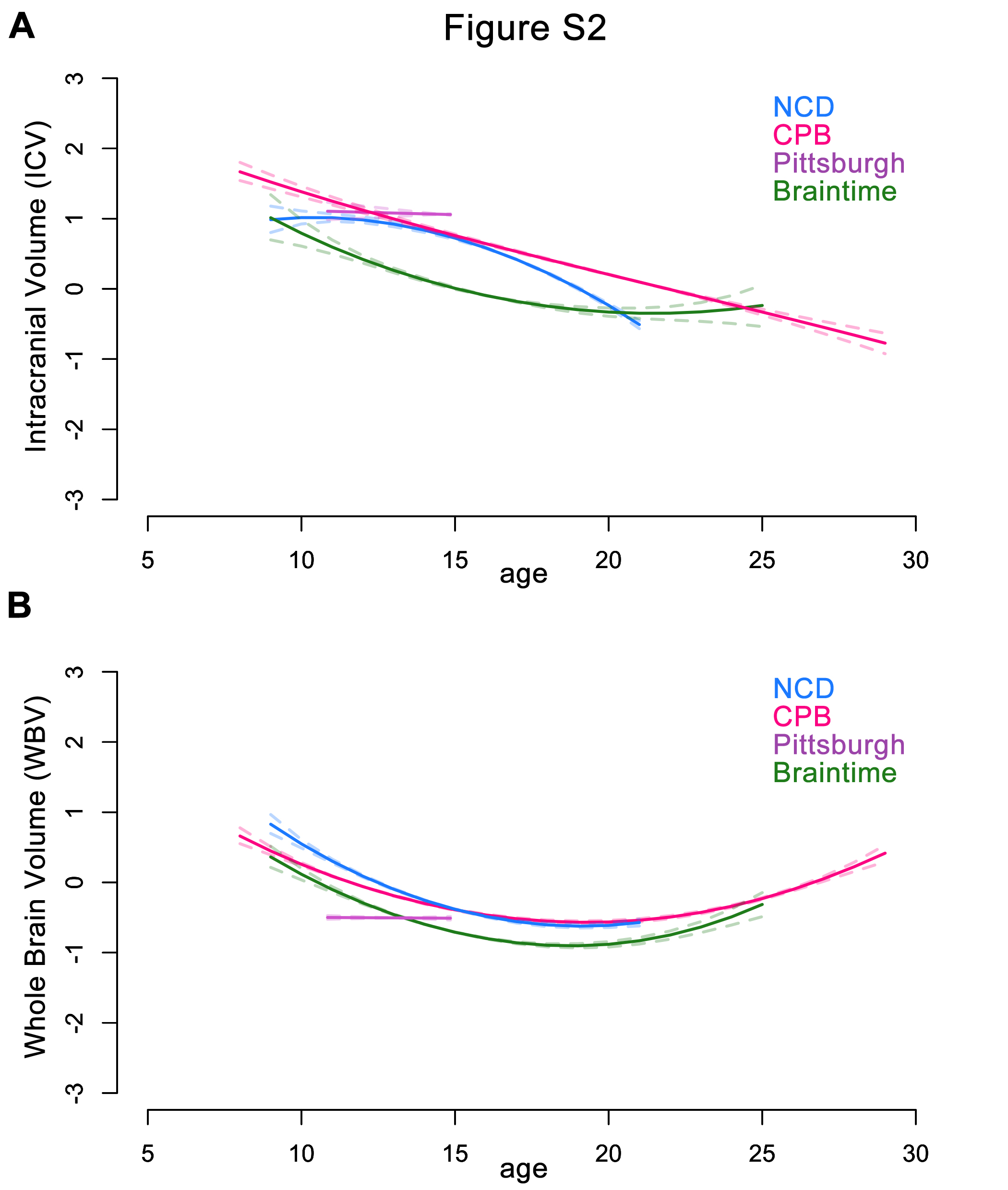
**

**Figure S2. Percent change models for Intracranial Volume (ICV) and Whole Brain Volume (WBV).** These graphs show the annual change in volume, as calculated based on the best fitting models for each of the four samples. a. ICV; b. WBV. ICV: Intracranial Volume; WBV: Whole Brain Volume; CPB: Child Psychiatry Branch; NCD: Neurocognitive Development.


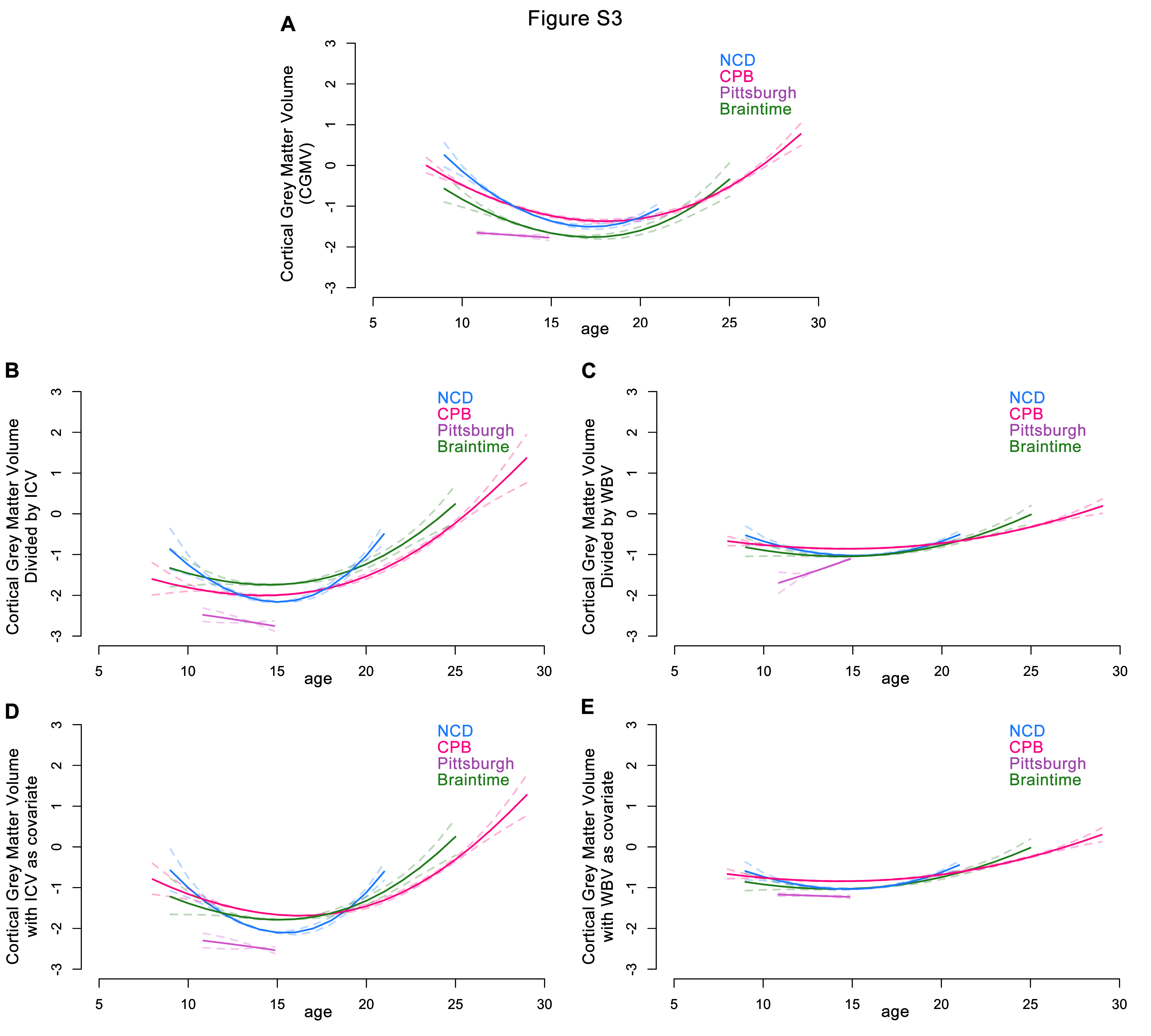


**Figure S3. Percent change models for Cortical Grey Matter Volume (CGMV).** These graphs show the annual change in volume, as calculated based on the best fitting models for each of the four samples. a. Raw values; b. CGMV adjusted by ICV (proportion); c. CGMV adjusted by WBV (proportion); d. CGMV with ICV included as a covariate; e. CGMV with WBV included as a covariate. ICV: Intracranial Volume; WBV: Whole Brain Volume; CPB: Child Psychiatry Branch; NCD: Neurocognitive Development.

**
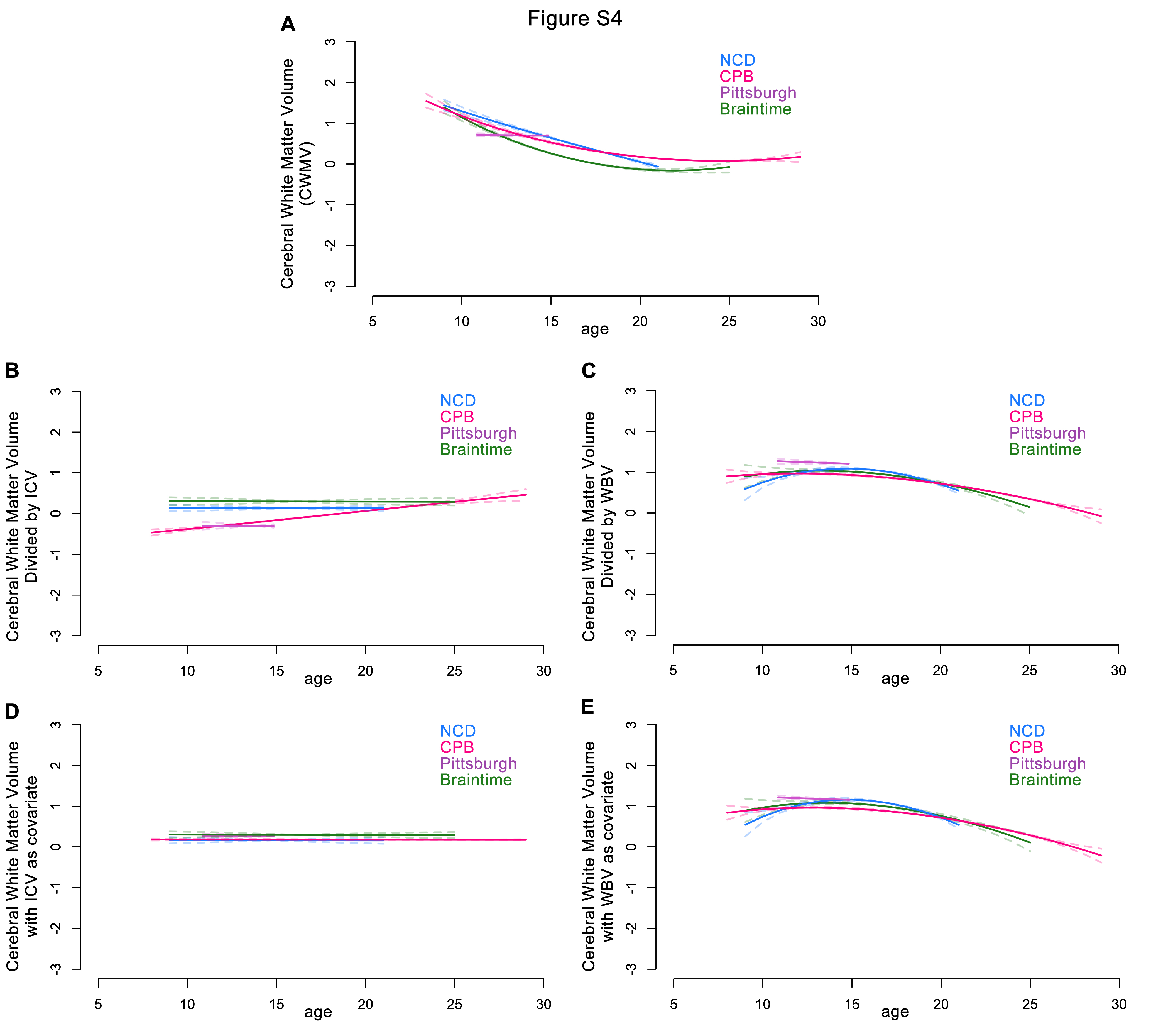
**

**Figure S4. Percent change models for Cerebral White Matter Volume (CWMV).** These graphs show the annual change in volume, as calculated based on the best fitting models for each of the four samples. a. Raw values; b. CWMV adjusted by ICV (proportion); c. CWMV adjusted by WBV (proportion); d. CWMV with ICV included as a covariate; e. CWMV with WBV included as a covariate. ICV: Intracranial Volume; WBV: Whole Brain Volume; CPB: Child Psychiatry Branch; NCD: Neurocognitive Development.


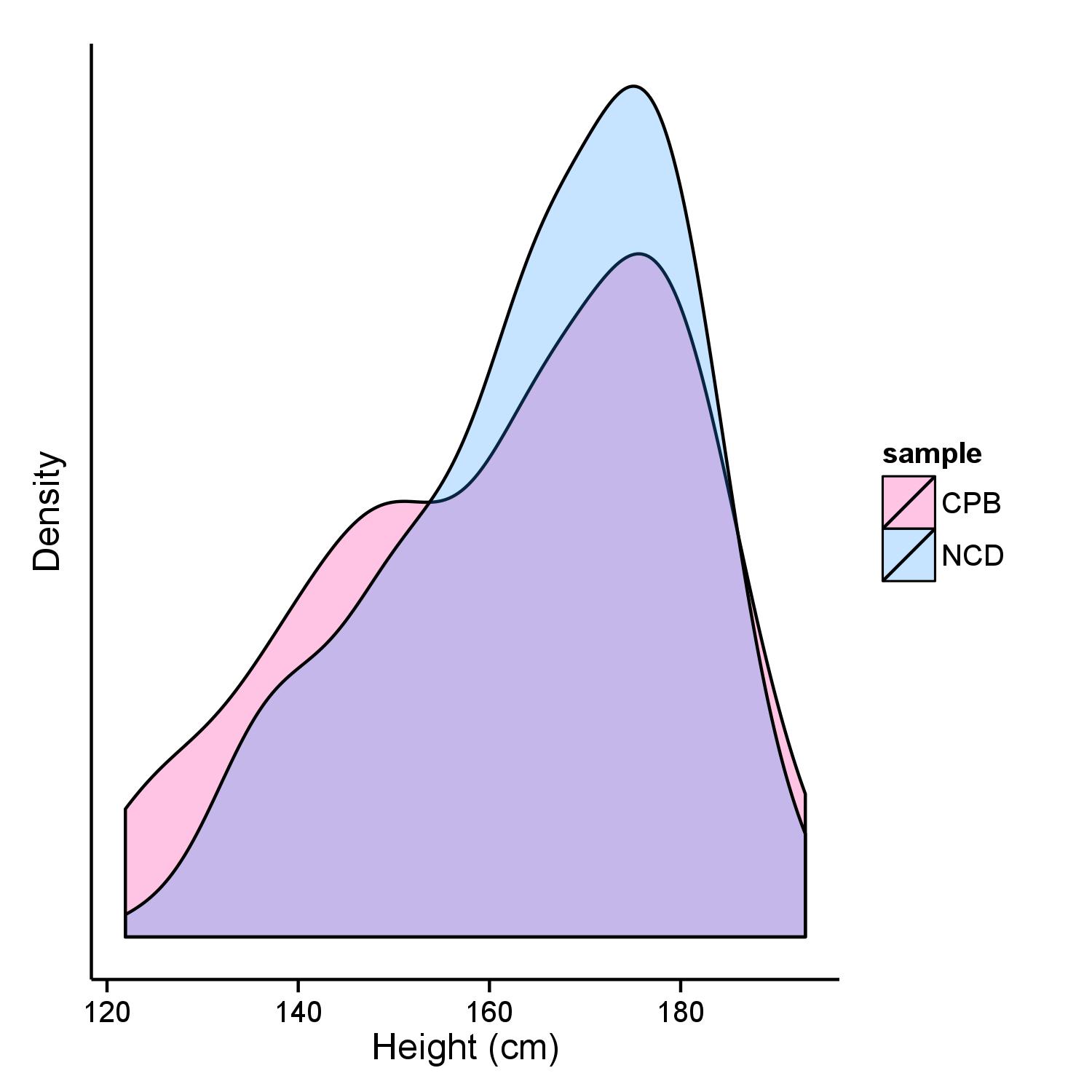


**Figure S5. Density plots of participant height in the Norwegian (NCD) sample and one of the US samples (CPB).** These graphs show the relative density of height (measured in cm) between the two samples that had longitudinal height information available for participants. These density plots show that more of the participants in the Norwegian sample (NCD) are at the taller end of the height spectrum than the participants in the US sample (CPB). cm: centimeter; CPB: Child Psychiatry Branch; NCD: Neurocognitive Development.


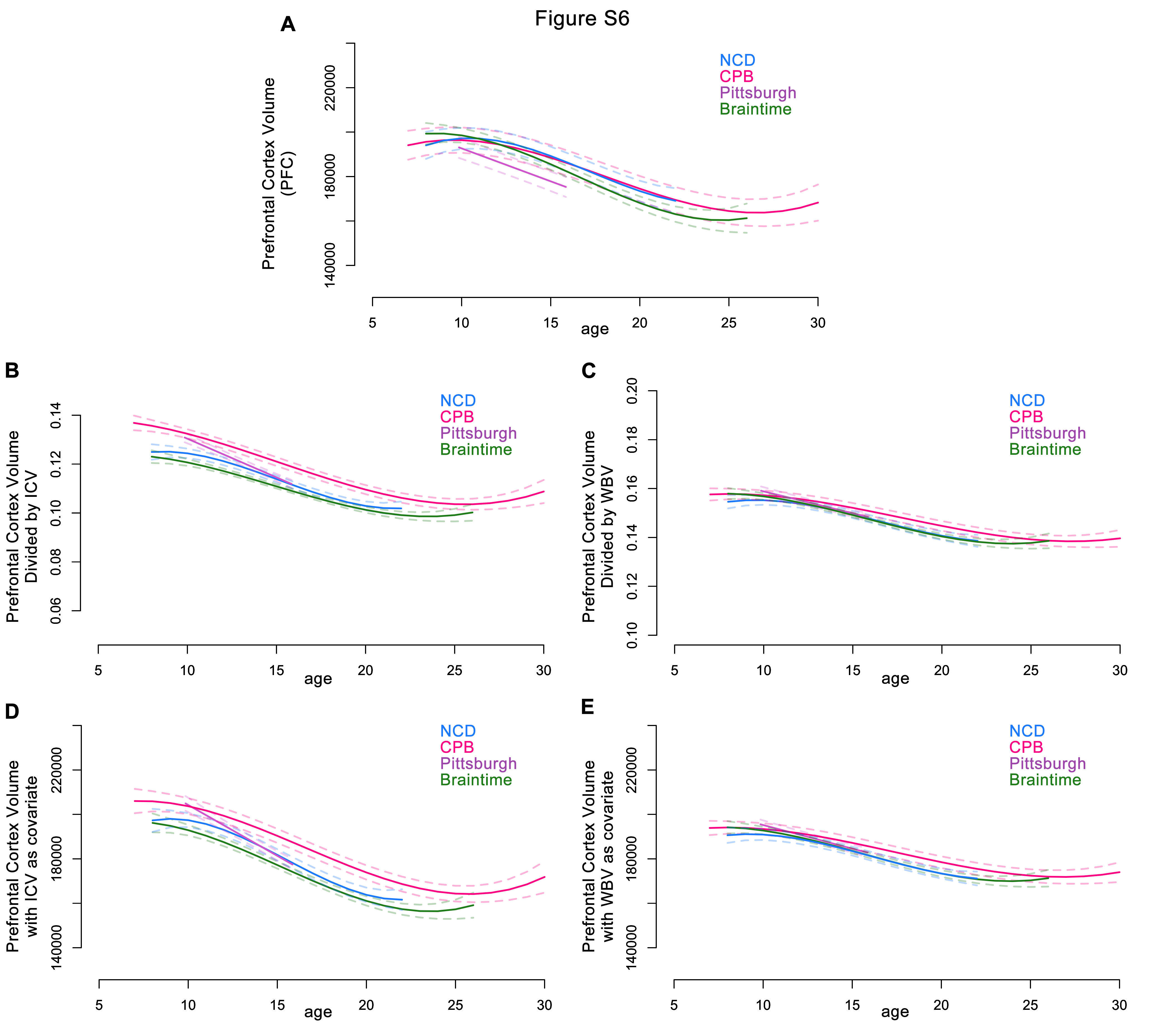


**Figure S6. Best fitting age models for Prefrontal Cortex Volume (PFC).** Age in years is measured along the x-axis and brain measure along the y-axis. a. Raw values (mm^3^); b. PFC adjusted by ICV (proportion); c. PFC adjusted by WBV (proportion); d. PFC with ICV included as a covariate (mm^3^); e. PFC with WBV included as a covariate (mm^3^). Best fitting models are represented by the solid lines. Dashed lines represent 95% confidence intervals. ICV: Intracranial Volume; WBV: Whole Brain Volume; CPB: Child Psychiatry Branch; NCD: Neurocognitive Development.


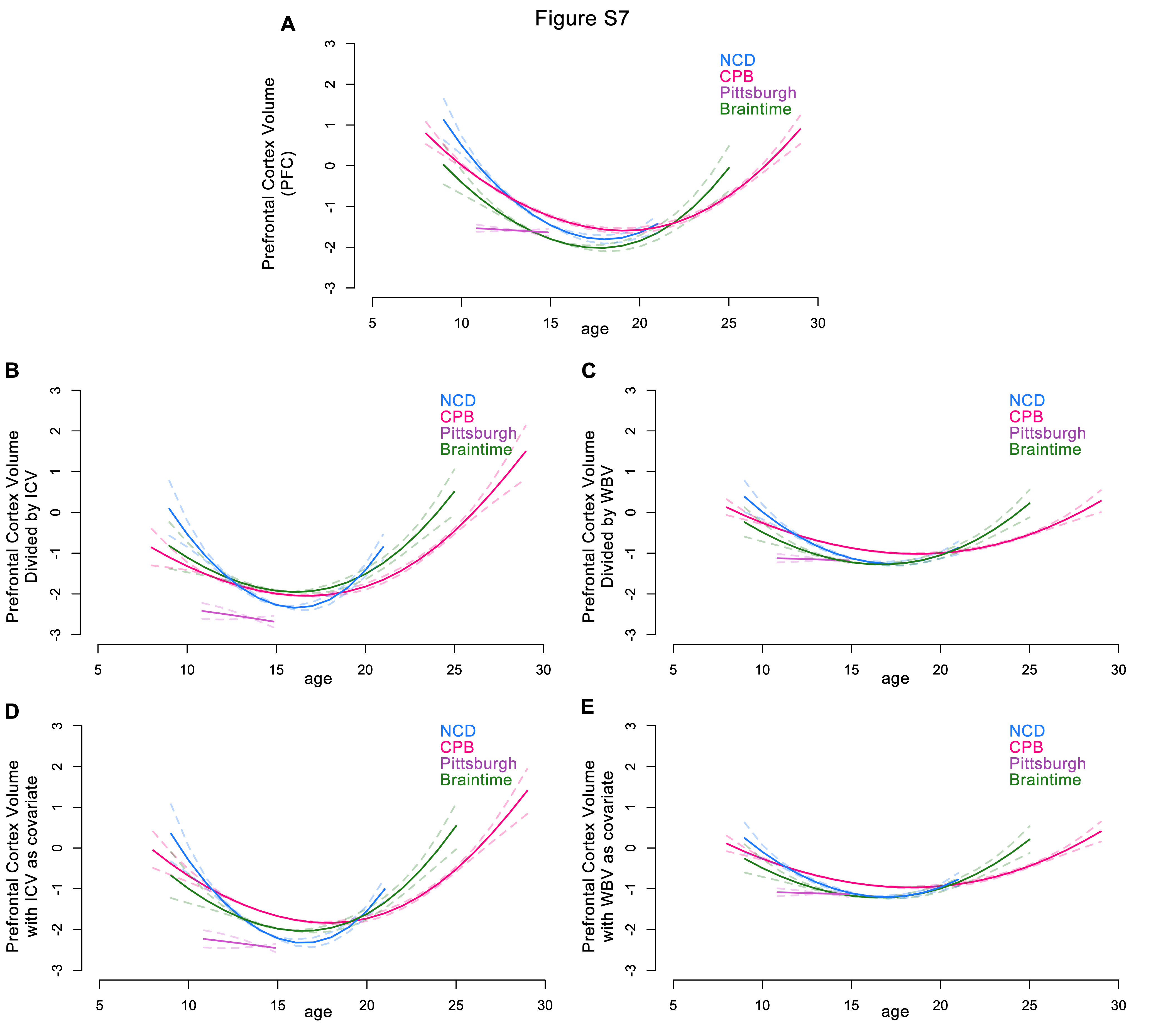


**Figure S7. Percent change models for Prefrontal Cortex Volume (PFC).** These graphs show the annual change in volume, as calculated based on the best fitting models for each of the four samples. a. Raw values; b. PFC adjusted by ICV (proportion); c. PFC adjusted by WBV (proportion); d. PFC with ICV included as a covariate; e. PFC with WBV included as a covariate. ICV: Intracranial Volume; WBV: Whole Brain Volume; CPB: Child Psychiatry Branch; NCD: Neurocognitive Development.

**Supplemental Experimental Procedures**

S1. Methods

S1.1 Participants

S1.1.1 CPB Sample

These 33 (10 female) participants were selected from a pool of over 1000 scans obtained by the NIMH Child Psychiatry Branch study of brain development (Giedd et al., 1996) for having fulfilled the criteria of having i) at least three high quality MRI scans spanning between late childhood and late adolescence, and ii) complete data on their physical development (height, weight, and pubertal status) at each time point (Mills et al., 2014). Participants were recruited from the community through local advertisement and were paid for their participation in the study. The institutional review board of the National Institutes of Health approved the research protocol employed in this study and written informed consent and assent to participate in the study were obtained from parents/adult participants and children respectively. Two individuals were monozygotic twins, and two pairs of individuals were siblings. Demographic characteristics were measured for each participant at the time of her or his first scan (see Table 1). These characteristics included ethnicity, socioeconomic status (SES; Hollingshead scales; Hollingshead, 1975), IQ (age-appropriate Wechsler Intelligence Scales; Wechsler, 1999) and handedness (Physical and Neurological Examination of Soft Signs inventory; Denckla, 1984). The IQs of participants in the sample ranged from 99–139 (mean 117.6 ± 10.5). There were no significant differences between females and males in handedness, ethnicity, IQ, SES, or number of scans. The absence of psychiatric disorder was established through completion of a screening questionnaire (Childhood Behavior Checklist; Achenbach and Edelbrock, 1991) at each time point.

S1.1.2 NCD sample

These participants were from a research project run by the Research Group for Lifespan Changes in Brain and Cognition, Department of Psychology, University of Oslo (Tamnes et al., 2013), approved by The Regional Committee for Medical and Health Research Ethics. Children and adolescents aged 8–19 years were recruited though newspaper advertisements and local schools. Written informed consent was obtained from all participants older than 12 years of age and from a parent of participants under 16 years of age, while participants under 12 years of age gave oral informed consent. At each time point, parents and participants aged 16 years or older complete screening for each participant with separate standardized health interviews to ascertain eligibility. Participants were required to be right-handed, be fluent Norwegian speakers, have normal or corrected-to normal vision and hearing, not have a history of injury or disease known to affect central nervous system (CNS) function, including neurological or psychiatric illness or serious head trauma, not be under psychiatric treatment, not use psychoactive drugs known to affect CNS functioning, not have had complicated or premature birth, and not have MRI contraindications. 76 participants (37 females) (see Table 1) satisfied these criteria and had adequate processed and quality-checked MRI data at two time points (9 individuals with scans from both time points were dropped due to low quality). Two individuals were dizygotic twins, and 9 pairs of individuals were siblings. The IQs of participants in the sample, as estimated by the Wechsler Abbreviated Scale of Intelligence (Wechsler, 1999), ranged from 87–141 (mean 109.6 ± 11.0) at the first time-point and 88–136 (mean 113.1 ± 10.2) at the second time-point.

S1.1.3 University of Pittsburgh Sample

These 126 typically developing adolescents, ages 10 to 14, were recruited through advertisements, flyers, and demographically targeted phone lists (Herting et al., 2014). The age range for this sample is limited to ages 10–14 years because this study was specifically designed to examine the effects of pubertal onset (versus age). Exclusionary criteria for all participants included a lifetime diagnosis of psychiatric disorders, irremovable metal, history of head injury, serious medical illness, or psychotropic medication. All participants and their legal guardians provided informed written consent to participate in the current study as approved by the University of Pittsburgh Institutional Review Board. Of the 126 recruited youth, 73 participants (41 girls) (see Table 1) were selected for the current study for having fulfilled the criteria of having two high quality MRI scans. Demographic characteristics were measured for each participant at the time of her or his first scan including ethnicity and handedness.

S1.1.4 Braintime Sample

The sample included 209 right-handed participants (112 females) between 8 and 24 years old (mean 14.7 ± 3.6), who were recruited through local schools and advertisements (Braams et al., 2015). At time point 1, 299 participants were scanned and 254 of them returned after two years for a second scan. Of these 254 participants, 209 participants had two high-quality scans (see Table 1). None of the participants reported a history of neurological or psychiatric disorders. All participants (or participant's primary caregivers for minors) provided written informed consent. Adults were paid for their participation, and minors and their primary caregivers received presents and a fixed payment for travel reimbursement. The internal review board from the Leiden University Medical Center approved the study procedures.

S1.2. Image Acquisition

S1.2.1 CPB Sample

All MRI scans were T-1 weighted images with contiguous 1.5 mm axial slices and 2.0 mm coronal slices, obtained on the same 1.5-Telsa General Electric Signa scanner (Milwaukee, WI) using a 3D spoiled gradient recalled echo sequence with the following parameters: repetition time (TR) 2,400 ms; echo time (TE) 5 ms; flip angle (FA) 45°; acquisition matrix 256 × 192; number of excitations 1; field of view (FOV) 24 cm; 124 axial slices; voxel size 0.9375 × 0.9375 × 1.5 mm. Each scan took 9 min 54 s. A clinical neuroradiologist evaluated all scans for gross abnormalities. These scans were processed using the same version of FreeSurfer (5.3), on the same operating system and workstation, utilizing the resources of the NIH HPC Biowulf cluster server (http://hpc.nih.gov).

S1.2.2 NCD Sample

MRI data were collected at using a 12-channel head coil on the same 1.5-Tesla Siemens Avanto scanner (Erlangen, Germany). The pulse sequence used for morphometric analyses was a 3D T1-weighted MPRAGE with the following parameters: TR 2,400 ms; TE 3.61 ms; inversion time (TI) 1,000 ms; FA 8°; acquisition matrix 192 × 192; FOV 240; 160 sagittal slices; voxel size 1.25 × 1.25 × 1.20 mm. The sequence was repeated at minimum twice in each session. Each scan took 7 min 42 s. A clinical neuroradiologist evaluated all scans for gross abnormalities. These scans were processed using the same version of FreeSurfer (5.3), and on the same operating system (RedHat Linux 64) and workstation.

S1.2.3 University of Pittsburgh Sample

All MRI scans were whole-brain T1-weighted MPRAGE images obtained on the same 3-Tesla Siemens Allegra scanner at the University of Pittsburgh. Scan parameters for the MPRAGE were as follows: TR 1,540 ms; TE 3.04 ms; FA 8°; FOV 256 × 256; voxel size 1.0 × 1.0 × 1.0 mm. A neuroradiologist evaluated all scans for gross abnormalities. These scans were processed using the same version of FreeSurfer (5.3), and on the same operating system (Ubuntu 12.04) and workstation.

S1.2.4 Braintime Sample

All participants were scanned on a 3-Tesla whole body Philips Achieva MRI system (Best, The Netherlands). High-resolution 3D T1-weighted anatomical scan were obtained: TR 9.75 ms; TE 4.59 ms; FA 8º; FOV 224 x 168 x 177.333; 140 slices; voxel size .8756 × .875 × 1.2 mm;). All anatomical scans were reviewed and cleared for gross abnormalities by a radiologist. These scans were processed using the same version of FreeSurfer (5.3), and on the same operating system (Linux Centos 6.3) and workstation.

S1.3 Post-hoc analysis of Prefrontal Cortex Volume

To address the concern that larger structures might be corrected for to different extent than smaller structures, we performed our same analyses on the prefrontal cortex (Figures S6 and S7). We defined the prefrontal cortex by combining the following parcels from the DKT parcellation atlas provided by FreeSurfer: caudal anterior cingulate, caudal middle frontal, lateral orbitofrontal, medial orbitofrontal, pars opercularis, pars orbitalis, pars triangularis, rostral anterior cingulate, rostral middle frontal, and superior frontal (Klein and Tourville, 2012). This analysis revealed similar sized effects in the prefrontal cortex as those found for the cortical grey matter volume analysis.

**Supplemental References**

Achenbach, T.M., Edelbrock, C., 1991. Child behavior checklist. Burlingt. Vt 7.

Braams, B.R., van Duijvenvoorde, A.C.K., Peper, J.S., Crone, E.A., 2015. Longitudinal changes in adolescent risk-taking: a comprehensive study of neural responses to rewards, pubertal development, and risk-taking behavior. J. Neurosci. Off. J. Soc. Neurosci. 35, 7226–7238. doi:10.1523/JNEUROSCI.4764-14.2015

Denckla, M., 1984. Revised Neurological Examination for Subtle Signs (1985). Psychopharmacol. Bull. 21, 773–800.

Giedd, J.N., Snell, J.W., Lange, N., Rajapakse, J.C., Casey, B.J., Kozuch, P.L., Vaituzis, A.C., Vauss, Y.C., Hamburger, S.D., Kaysen, D., Rapoport, J.L., 1996. Quantitative magnetic resonance imaging of human brain development: ages 4-18. Cereb. Cortex N. Y. N 1991 6, 551–560.

Herting, M.M., Gautam, P., Spielberg, J.M., Kan, E., Dahl, R.E., Sowell, E.R., 2014. The role of testosterone and estradiol in brain volume changes across adolescence: a longitudinal structural MRI study. Hum. Brain Mapp. 35, 5633–5645. doi:10.1002/hbm.22575

Hollingshead, A.B., 1975. Four Factor Index of Social Status.

Klein, A., Tourville, J., 2012. 101 labeled brain images and a consistent human cortical labeling protocol. Front. Brain Imaging Methods 6, 171. doi:10.3389/fnins.2012.00171

Mills, K.L., Goddings, A.-L., Clasen, L.S., Giedd, J.N., Blakemore, S.-J., 2014. The developmental mismatch in structural brain maturation during adolescence. Dev. Neurosci. doi:10.1159/000362328

Tamnes, C.K., Walhovd, K.B., Dale, A.M., Østby, Y., Grydeland, H., Richardson, G., Westlye, L.T., Roddey, J.C., Hagler, D.J., Jr, Due-Tønnessen, P., Holland, D., Fjell, A.M., 2013. Brain development and aging: Overlapping and unique patterns of change. NeuroImage 68C, 63–74. doi:10.1016/j.neuroimage.2012.11.039

Wechsler, D. 1896-, 1999. WASI Wechsler abbreviated scale of intelligence, Wechsler abbreviated scale of intelligence. Psychological Corporation, Hove.
